# Drug Habit

**Published:** 1915-11

**Authors:** 


**Drug Habit.** Joseph C. Doane of the Phila. Gen. Hosp., gives the following reasons for establishment of opium or other drug habit. Of the twenty-three cases in which smoking opium was given, association was really the starting point, upon the invitation of friends or from curiosity.

| | |
|---|---|
| Association | 86 |
| Doctor's prescription | 14 |
| Smoking opium | 23 |
| Other drugs, mainly cocain | 4 |
| To keep awake | .2 |
| To cause sleep or quiet nerves | 17 |
| Not stated | .7 |
| Total | 153 |

